# The Impact of PLCG2*M28L and Diet on Neuro‐metabolic and Vascular Dysfunction in Novel Mouse Models of Late‐Onset Alzheimer's Disease

**DOI:** 10.1002/alz70856_098702

**Published:** 2025-12-24

**Authors:** Juan Antonio Kim Hoo Chong Chie, Scott A Persohn, Adrian L Oblak, Kierra Eldridge, Kevin P Kotredes, Ravi S Pandey, Stacey J Sukoff Rizzo, Gregory W Carter, Michael Sasner, Gareth R Howell, Bruce T. Lamb, Paul R Territo

**Affiliations:** ^1^ Stark Neuroscience Research Institute, Indiana University School of Medicine, Indianapolis, IN, USA; ^2^ Stark Neurosciences Research Institute, Indiana University School of Medicine, Indianapolis, IN, USA; ^3^ The Jackson Laboratory, Bar Harbor, ME, USA; ^4^ University of Pittsburgh School of Medicine, Pittsburgh, PA, USA

## Abstract

**Background:**

MODEL‐AD has developed and characterized novel mouse models that aim to better phenocopy human LOAD for preclinical testing. More than 40 different models carrying combinations of LOAD risk factors have been created and assessed for relevance to LOAD. In this study, we aim to determine the mechanisms by which variations in the phospholipase C gamma 2 (*PLCG2*) gene drives the increase risk for LOAD with or without high‐fat diet (HFD).

**Method:**

The *M28L* variant in *PLCG2* (PLCG2*M28L) was prioritized as a LOAD risk factor. Mice were generated by CRISPR/CAS9 introduction of Plcg2*M28L variant on to LOAD2 (B6.*APOE4*. Trem2*R47H.hAβ) and transcriptional profiled at 18 months. To evaluate the contribution of LOAD risk, LOAD2 and LOAD2.Plcg2*M28L mice were fed control or HFD as a driver of neuroinflammation. Female and male mice were characterized at 18 months via the MODEL‐AD phenotyping pipeline. Uncoupling and connectomics analysis of *in vivo* brain perfusion and glycolytic metabolism via PET/CT were performed.

**Result:**

Uncoupling analysis of LOAD2 on HFD and LOAD2.Plcg2*M28L mice both showed a prodromal (↑18F‐FDG,↑CBF) phenotype relative to LOAD2 mice. However, when LOAD2.Plcg2*M28L were fed HFD, they showed Type 2 (↑18F‐FDG,↓CBF) uncoupling phenotype relative to LOAD2.Plcg2*M28L mice. This suggests that the combination of risk factors is not simply additive. Multi‐resolution connectomics revealed a difference in clusters numbers and organization between male and females. LOAD2.Plcg2*M28L on HFD relative to LOAD2.Plcg2*M28L showed fewer clusters compared to LOAD2 on HFD and LOAD2.Plcg2*M28L mice. In addition, significant changes in clustering coefficient, positive nodal strength, and negative nodal strength were found. Region set enrichment analysis in LOAD2 on HFD and LOAD2.Plcg2*M28L mice showed similar phenotypic changes; however, LOAD2.Plcg2*M28L on HFD relative to LOAD2.Plcg2*M28L showed a reduction in modules and different endophenotypes.

**Conclusion:**

Data collected to date revealed that *Plcg2* and HFD have a similar effect on the end biology of the brain. Moreover, this suggests *Plcg2* is involved in an inflammatory process in a similar manner to HFD; however, *Plcg2* in the presence of HFD showed to be responsive to the diet effect in a regulatory manner which reduced disease stage.

